# Supplementary figures and images for: The glycosomal ATP-dependent phosphofructokinase of Trypanosoma brucei operates also in the gluconeogenic direction
Source: PLoS Biol. 2025 May 16;23(5):e3002938. doi: 10.1371/journal.pbio.3002938 (PMC12121924; doi:10.1371/journal.pbio.3002938)

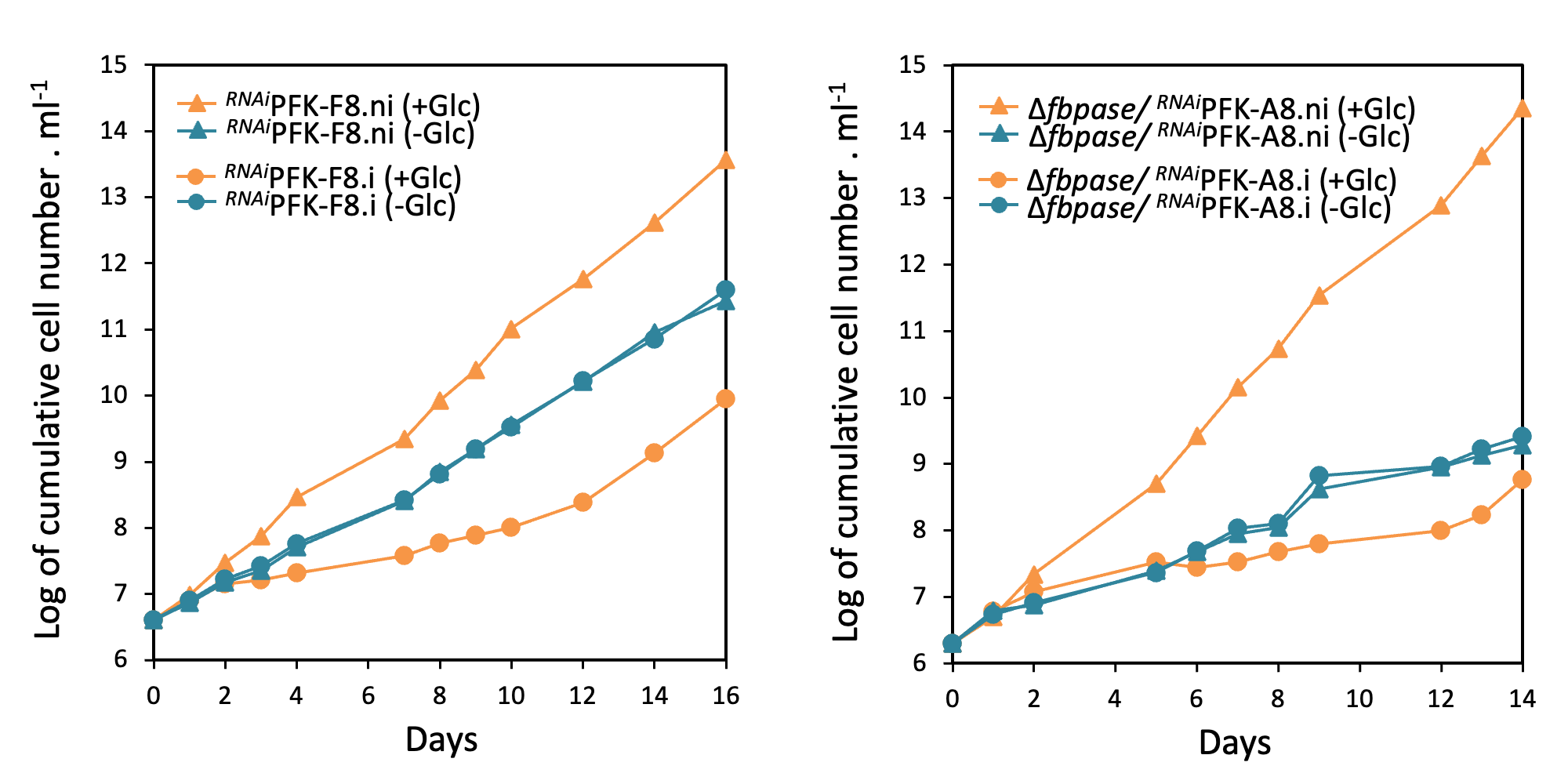

Supplement: S1 Fig — This figure shows growth curves of the RNAiPFK clone A8 and Δfbpase/RNAiPFK clone F8 mutant cell lines, tetracycline-induced or not, in glucose-rich (+Glc) and glucose-depleted (−Glc) conditions. Cells were maintained in the exponential growth phase (between 106 and 107 cells ml−1), and cumulative cell numbers reflect normalization for dilution during cultivation. Similar growth curves were obtained for the RNAiPFK clone A4 and Δfbpase/RNAiPFK clone A9 presented in Fig 3B. The data underlying this figure can be found in https://doi.org/10.5281/zenodo.15148560. (TIF) [file pbio.3002938.s001.tif]

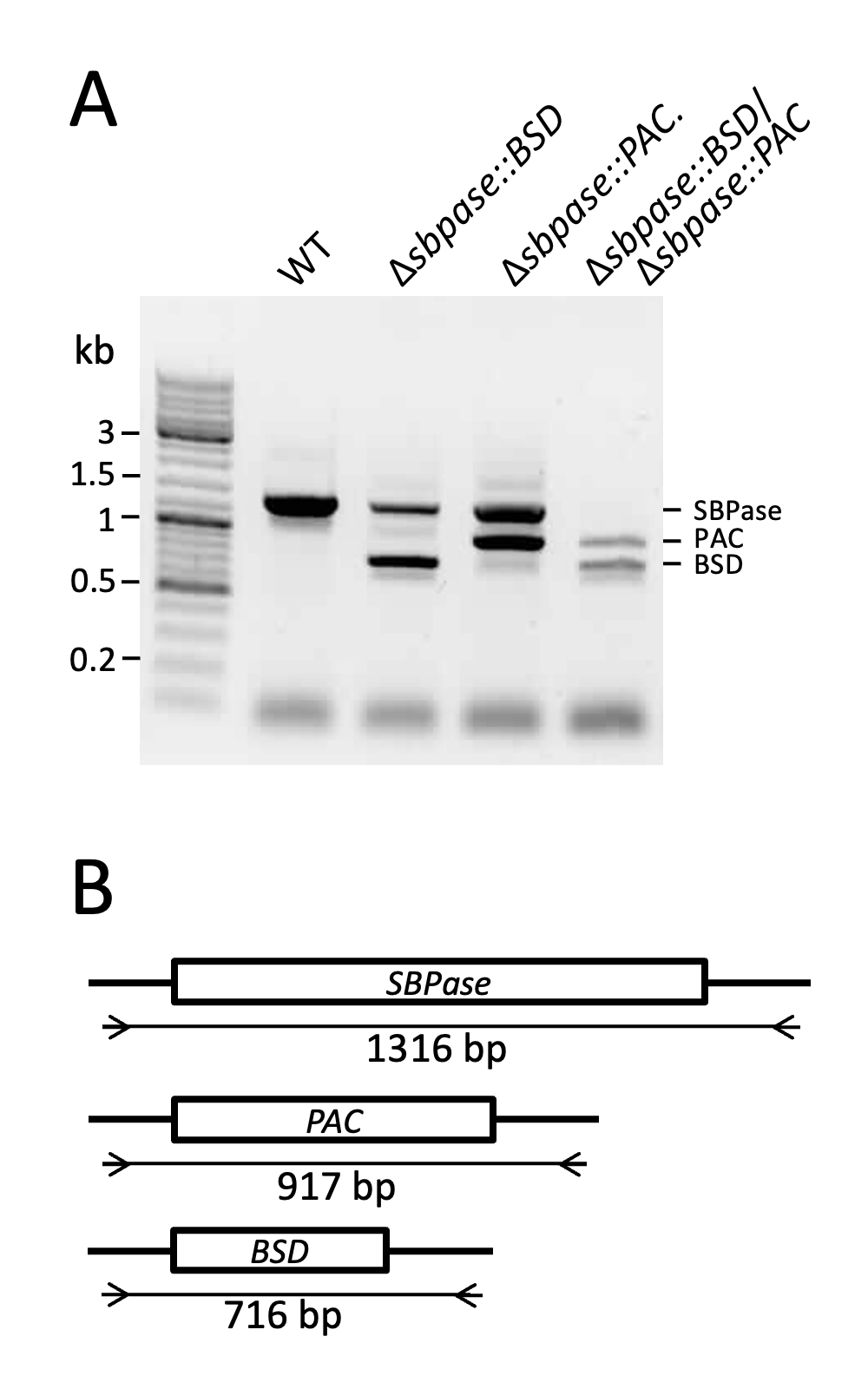

Supplement: S2 Fig — Panel A shows a PCR analysis of genomic DNA isolated from the parental (WT), Δsbpase::BSD, Δsbpase::PAC and Δsbpase::BSD/Δsbpase::PAC (named Δsbpase in the manuscript) cell lines, using two primers flanking the SBPase gene (5′-ggaggtactttctcttctatttct-3′ and 5′-aagtcagagcacattacgaccac-3′). The 3 PCR products, SBPase, PAC, and BSD, correspond to the DNA fragments described in panel B. As expected, PCR amplification of the SBPase gene was not observed in Δsbpase::BSD/Δsbpase::PAC cell ine, BSD PCR-products were observed in the Δsbpase::BSD and Δsbpase::BSD/Δsbpase::PAC cell lines, whereas PAC PCR-products were observed in the Δsbpase::PAC and Δsbpase::BSD/Δsbpase::PAC cell lines. The data underlying this figure can be found in https://doi.org/10.5281/zenodo.15148560. (TIF) [file pbio.3002938.s002.tif]
