# Supplementary material for: The glycosomal ATP-dependent phosphofructokinase of Trypanosoma brucei operates also in the gluconeogenic direction
Source: PLoS Biol. 2025 May 16;23(5):e3002938. doi: 10.1371/journal.pbio.3002938 (PMC12121924; doi:10.1371/journal.pbio.3002938)
Supplement: Attachment — Submitted filename: Response to reviewers.pdf [file pbio.3002938.s005.pdf]

We would like to thank the two reviewers for their valuable comments and corrections, which considerably improved the quality of our manuscript.

Thank you for your patience while your manuscript "The glycosomal ATP-dependent phosphofructokinase of Trypanosoma brucei operates in the gluconeogenic direction in cellulo under in vivo-like conditions" went through peer-review at PLOS Biology. Your manuscript has now been evaluated by the PLOS Biology editors, an Academic Editor with relevant expertise, and by several independent reviewers.

As you will see in the reports, the reviewers are generally positive but have raised some concerns and provided suggestions for improvement. Reviewer #1 requests clarifications on experimental methods and the inclusion of missing statistical tests. Notably, it is unclear how many replicates were performed for each experiment. If the data represent repeats, please specify this in the text; however, if they are from a single replicate, additional experiments will be necessary to ensure robustness.

Answer: We have addressed all the comments raised by reviewer #1 (see below).

Additionally, Reviewer #2 suggests incorporating dynamic models to evaluate various scenarios.

Answer: Indeed, mathematical models of glycolysis, based on all available kinetic data previously obtained for glycolytic/gluconeogenic enzymes, have been previously produced to possibly study any interplay between gluconeogenesis versus glycolysis under different conditions. Unfortunately, the currently available models are based on bloodstream form trypanosomes (BSF), which have a significantly different glycosomal metabolism compared to procyclic trypanosomes (PCF). We consider that the BSF model is not appropriate to address the very interesting point raised by reviewer #2 (see our specific answer below). The construction of a model based on the PCF glycosomal metabolism is a project in itself, which cannot be carried out in the time frame allotted to the revision of the manuscript.

We expect to receive your revised manuscript within 2 months. Please email us (plosbiology@plos.org) if you have any questions or concerns, or would like to request an extension.

At this stage, your manuscript remains formally under active consideration at our journal; please notify us by email if you do not intend to submit a revision so that we withdraw the manuscript.

— — —

Reviewer #1:

The Authors found that, using glycerol as a carbon source, T. brucei procyclic forms produce fructose 6-phosphate from fructose 1,6-bisphosphate using fructose 1,6-bisphosphatase, sedoheptulose 1,7-bisphosphatase, and ATP-dependent phosphofructokinase (PFK). The function of PFK in gluconeogenesis rather than in glycolysis is novel and attributed to the supramolecular organization of the pathway within glycosomes that allows metabolic channeling and produces ATP to maintain the ATP/ADP glycosomal balance. This is a thoughtfully considered study. Overall, this manuscript could advance the field, but some clarifications are needed.

1. Results, (line 168). The paragraph starts with the description of previous results, but it is not clear whether it continues with the description of those results, or it starts describing new results ("This was determined…": when, before or in this work?). This section needs a better description of Fig. 2 to explain how the conclusions were reached considering what the figure is showing.

Answer: Indeed, Data obtained with [U-^{13}C]-proline have previously been presented in Wargnies *et al.* PLoS Pathogen, 2018 (ref 23). They have been included in Figure 2 for comparison with the labelling with [U-^{13}C]-glycerol. Two sentences have been included in the first paragraph of the results section, "These data, previously described in [23] and included in Figure 2 (columns 4-5, labelling with [U-^{13}C]-proline), demonstrated that FBPase is involved in gluconeogenesis" and in the figure legend "Data obtained with [U-^{13}C]-proline, previously presented in Wargnies *et al.* [23], have been included here for comparison with the labelling with [U-^{13}C]-glycerol".
In addition, the first paragraph of the results section, as well as figure 2 (see below), have been modified to better describe the data presented in this figure.

It is not clear to me where it is shown in Fig. 2 that 91% of F1,6BP were fully [13C]-enriched from [U-13C] glycerol, compared to 48% from [U-13C] proline.

Answer: This has been clarified by explicitly mentioning the corresponding columns and the cell line in the main text (in red), "As expected, 91.0 ±2.2% of F1,6BP in the parental cells were fully [^{13}C]-enriched (m6) from [U-^{13}C]-glycerol, compared to 48.5 ±5.9% from [U-^{13}C]-proline (Fig 2; F1,6BP; columns 1 *vs* 4)".
In additions, we have included the following sentence in the figure legend, "Each value corresponds to the mean of 3 independent experiments (biological replicates) and a t-test was performed by assuming equal variances to determine the p-value (*, $p < 0.1$; **, $p < 0.01$; ***, $p < 0.001$; ****, $p < 0.0001$; ns, not significant)".

The meaning of the different arrows in the figure should be indicated.

Answer: The colour of the arrows has been changed to make it easier to understand the direction of metabolic flux depending on the carbon source used, as explained in the revised figure legend, "The colour of the arrows corresponds to metabolism of glycerol (blue), proline (orange) or the two ^{13}C-enriched carbon sources (black). The solid and dotted arrows correspond to a single or at least 2 enzymatic steps".

What are the other bands that are not due to PFK?

Answer: Unfortunately, the anti-PFK immune serum, provided by Paul Michels' lab, is not specific to PFK with additional unknown proteins detected. However, the band absent in the PFK null mutant corresponds to a protein with an apparent molecular weight compatible with PFK (54 kDa).

The western blot needs some indication of the MW of the bands and the full blot should be presented in a supplementary figure. The same needs to be done with all other western blot analyses in the paper (Figs. 3A, 4A, 5C, 6C, 7A and 9H).

Answer: The position of molecular weight markers is indicated in all these western blots.

In addition, when quantifications of western blot results are presented, the Authors should provide information about the number of western blots (n), and the statistical treatment of the data.

Answer: For the western blot in Figure 7, three to five biological replicates have in included in the statistical analysis per sample. Details of the analyses were compiled in the metadata file attached to the submission. The following sentences has been included in the figure legend "Each value corresponds to the mean (±SD) of 3 to 5 independent experiments (biological replicates) and a Student

t-test was performed by assuming equal variances to determine the p-value (*, $p < 0.1$; **, $p < 0.05$; ***, $p < 0.01$; ****, $p < 0.005$)".

2. Why Fig. 3B does not show s.d.? Is it only one experiment?

Answer: The growth curve of this cell lines (^{RNAi}PFK clone A4, Δ*fbpase*/^{RNAi}PFK clone A9) have been done at least 2 times. Unfortunately, these ^{RNAi}PFK cell lines are instable with significant re-expression of PFK allowing restoring growth of the parasite. However, we have analysed other ^{RNAi}PFK (A8) and Δ*fbpase*/^{RNAi}PFK (F8) clones, which show the same growth pattern as observed for those included in Figure 3B. It is noteworthy that the growth phenotype reversion is observed in the ^{RNAi}PFK clone A8 and Δ*fbpase*/^{RNAi}PFK clones F8 after 13 days of tetracycline-induction. These data have been included in the supplementary Figure S1, and the following sentence was included in the figure legend: "Similar growth curves were obtained for the ^{RNAi}PFK clone A8 and Δ*fbpase*/^{RNAi}PFK clone F8 (Fig. S1)".

Fig. 3C shows error bars with no indication of what they are, the number of experiments (n) and the statistical test used to detect significant differences.

Answer: the following sentence was included in figure legend, "Each value corresponds to the mean of 3 independent experiments (biological replicates) and a t-test was performed by assuming equal variances to determine the p-value (****, $p < 0.0001$; *****, $p < 0.00001$)"

Fig. 3D needs clarification regarding how the % enrichment is evident when seeing the figure.

Answer: In Figure 3D, as well as in Figures 2 and 4C, a colour code is used to distinguish between molecules containing from 0 to 6 ^{13}C-enriched carbons. This colour code is indicated in each figure (from dark blue to orange for M0 to M6, respectively. To clarify this representation, the colour code is explicitly mentioned in each figure, "The figure shows enrichment of key glycolytic intermediates at 0 to 6 carbon positions (m0 to m6, with a colour code on the side) with [^{13}C]-labelled atoms expressed as percentage of all corresponding molecules (MID; Mass Isotopomer Distribution)".
We have also included a sentence introducing this section, "The role of PFK in gluconeogenesis was determined by IC-MS/MS analyses of the incorporation levels of ^{13}C atoms from metabolism of [U-^{13}C]-glycerol] into key gluconeogenic intermediates, in the parental, *Δfbpase*, ^{RNAi}PFK (.ni and .i) and Δ*fbpase*/^{RNAi}PFK (.ni and .i) cell lines (Fig. 3D)".

3. Fig. 4A. The first arrow does not indicate any band.

Answer: The arrow has been repositioned to show the correct band.

Where is the genetic evidence of SBPase KO?

Answer: We have included in the supplementary data (Fig. S2) the genetic evidence of deletion of both alleles of the *SBPase* gene, using the gene replacement approach.

4. Does Fig. 5E show the results of a single experiment?

Answer: These growth curves have been produced in triplicate. The figure was modified by including the mean and SD of the three experiments.
To be consistent we have done the same modification for figure 4B.

5. Fig. 8 needs to indicate meaning of error bars, number of experiments (n).

Answer: Each value corresponds to the mean of 3 independent experiments (biological replicates). This sentence was added in the figure legend.
Similarly, the same sentence was included in the legend of figures 2, 3D, 4C, corresponding to HRMS data, with 3, 3 and 4 biological replicates, respectively.

6. Fig. 9I and 9J need to indicate meaning of error bars, number of experiments (n), significance and statistical test used to detect significant differences.

Answer: The number of independent experiments for each condition was indicated in the figure legend (i.e. "means ±SD, n=3 independent experiments"). A Student t-test was performed by assuming equal variances to determine the p-value and included in the figure. The following sentences has been included in the figure legend "Each value corresponds to the mean (±SD) of 3 to 5 independent experiments (biological replicates) and a Student t-test was performed by assuming equal variances to determine the p-value (*, $p < 0.1$; **, $p < 0.05$; ***, $p < 0.01$; ****, $p < 0.005$: ns, not significant)".

7. Lines 729-730 refer to something underlined and in italics. What figures shows that?

Answer: The short sequence (<u>TAG</u>C<u>TAA</u>G<u>TGA</u>*ggatcc*<u>TGA</u>A<u>TAA</u>T<u>TAG</u>) mentioned in the same sentence contains stop codons (underlined) and a *Bam*HI restriction site (italic letters). We have added "in the short sequence above" after "underlined" and "italic letters".

8. Line 875. Are there video-microscopy experiments?

Answer: We have removed "video-", since there is no video presented in the manuscript.

— — —

Reviewer #2:

The article of Plazolles et al. is a rich depiction of contributions of various enzymes to gluconeogenesis in the procyclic form of Trypanosoma brucei. It breaks the dogma that ATP-dependent phosphofructokinases cannot be used in GNG and simultaneously provides new information on contributions of sedoheptulose bisphosphates and fructose bisphosphatase. The work includes an extraordinarily rich diversity of approaches including IC-MS based metabolic profiling and NMR analysis of metabolic end products. An impressive array of multiple gene knockouts/knockdowns, exploitation of marker free CRISPR-cas approaches and novel routes to ascertain concentrations of ATP/ADP in cellulo.

There are just a couple of things I would like to have a clearer view upon.

Firstly it is the physiological role of PFK in GNG in trypanosomes. Could a sentence be added clarifying why glycerol is so much better than proline as a substrate for study (other than its better incorporation in H6P. It would be useful to explicitly outline why that is so.

Answer: To explain why glycerol is a better gluconeogenic substrate than proline, the following sentence has been included before describing the data, "Indeed, for the same incubation time in the presence of the ^{13}C-carbon source (2h), [U-^{13}C]-glycerol is directly incorporated into gluconeogenic intermediates (see Fig 1), whereas incorporation of [U-^{13}C]-proline into gluconeogenic intermediates may be significantly delay by the 9-step metabolic pathway, including 7 mitochondrial steps, leading to PEP from proline [14]".

Presumably proline would be the physiologically relevant substrate in tsetse fly (although might glycerol be more abundant than hitherto believed in tsetse, for instance via their fat bodies?).

Answer: Unfortunately, no data are yet available on the quantities of glycerol in the midgut of the tsetse fly, which makes it difficult to speculate on the possible role of glycerol in vivo in the insect vector, in particular to feed gluconeogenesis. Anyway, we have added a sentence in the first paragraph of the discussion section suggesting that a possible presence of glycerol in the digestive tract would facilitate gluconeogenesis in the insect midgut, "It is noteworthy that the possible presence of glycerol in the insect midgut would significantly stimulate gluconeogenesis, however, no *in vivo* data are yet available to validate this hypothesis."

Significant and effective models for glycolytic flux have been generated for bloodstream form trypanosomes. I appreciate that the current paper is not dependent upon modelling, but have the authors contemplated exploiting dynamic models of metabolism to ascertain a spectrum of scenarios around PFK working in either direction and consequences of titrating alternative starting substrated to probe any interplay between GNG vs glycolysis under different conditions?

Answer: Indeed, mathematical models of glycolysis, based on all available kinetic data previously obtained for glycolytic/gluconeogenic enzymes, have been previously produced to possibly study any interplay between gluconeogenesis versus glycolysis under different conditions (ref). Unfortunately, the currently available models are based on bloodstream form trypanosomes (BSF), which have a significantly different glycosomal metabolism compared to procyclic trypanosomes (PCF). This is particularly important in the context of the direction of PFK activity, which may depend on the balance of the glycosomal ATP/ADP ratio, since the enzymes used to maintain this balance differ between BSF and PCF. Indeed, the glycosomal PGK required to balance the glycosomal ATP/ADP ratio in BSF, is no longer expressed in PCF, which express instead PPDK and PEPCK, the latter being part of 4-step glycosomal pathway leading to succinate production. It is also important to consider that glycolytic flux is 10 times higher in BSF than in PCF. In this context, we consider that the BSF model is not appropriate to address the very interesting point raised by the reviewer. The construction of a model based on the PCF glycosomal metabolism is a project in itself, which cannot be carried out in the time frame allotted to the revision of the manuscript.

Given the dogma-busting nature of the discovery of a PFK operating in GNG, some contemplation on whether this reflects the evolutionary history of the trypanosome's enzyme. If it evolved from a PPi dependent PFK is it possible it had an ancestral role in GNG, then its switch to use of ATP may have been possible due to the regulatory environment and possible concentration differentials within different sub-compartments (e.g. tunneling within an enzyme complex) that has permitted the change. What might expressing a PPi dependent PFK in trypanosomes do? (I'm not advocating for this experiment to be done here, just curious).

Answer: A recent phylogenetic analysis of PPi-dependent PFK and ADP-dependent PFK from organisms belonging to phylum Euglenozoa, including kinetoplastidea, diplonemea and euglenida classes, suggested that PFK ('clade X') of the common Euglenozoa ancestor, retained in the trypanosomatid lineages, was almost certainly ATP dependent, indicating that phospho-donor specificity had already changed from PPi to ATP well before the ancestral Euglenozoan (Andrade-Alviarez, 2022). Thus, it seems reasonable to consider that the trypanosomatid ancestor never expressed a PPi-dependent glycosomal PFK, since glycosomes appeared in the euglenida class (Andrade-Alviarez, 2022), after the transition from PPi to ATP dependence. In this context, replacing the ADP-dependent PFK by the PPi-dependent enzyme in procyclic trypanosomes, is of interest, but would not provide a relevant answer for the acquisition of the gluconeogenic role of ADP-dependent PFK.

A paragraph describing this situation has been included in the discussion section, “Interestingly, the trypanosomatid ATP-dependent PFK belongs to a group consisting predominantly of PPi-dependent PFK, suggesting that the trypanosome ancestor expressed a PPi-dependent PFK with a possible gluconeogenic role, which changed its phospho-donor specificity [16]. However, a recent phylogenetic analysis of PPi-dependent and ADP-dependent PFK from organisms belonging to phylum Euglenozoa, including Kinetoplastidea, Diplonemea and Euglenida classes, suggested that PFK (‘clade X’) of the common Euglenozoa ancestor, retained in the trypanosomatid lineages, was almost certainly ATP dependent, indicating that phospho-donor specificity had already changed from PPi to ATP well before the ancestral euglenozoan [53]. Consequently, the trypanosomatid ancestor probably never expressed a PPi-dependent glycosomal PFK, since glycosomes (*i.e.* peroxisomes having acquired glycolytic/gluconeogenic enzymes) are only found in kinetetoplastids and diplonemids, not euglenids, thus must have appeared in the Euglenozoa class [53] only after the transition from PPi to ATP dependence”.

line 33: add comma after "through gluconeogenesis"
Answer: The comma was added

line 35: changed "showed" to "show"

Answer: This change has been made

Line 58: Remove "the" before "central carbon metabolism"

Answer: “The” was removed

line 60: change to "tightly regulated allosterically"

Answer: This change has been made

line 79: remove "the" before "kidney"

Answer: “The” was removed

Line 87: humans (with an "s")

Answer: The “s” was added

line 102: "by analogy" better than "in analogy"

Answer: This change has been made

line 104: put commas around "for instance"

Answer: The commas were added

line 107: remove "the" before PCF

Answer: “The” was removed

Fig 1 title, also remove "the" before PCF

Answer: “The” was removed

line 139: add "the" before PPP

Answer: “The” was included

line 143: remove "the" before cofactor and add "the" before PPP

Answer: These changes have been made

line 145: E4P (not EAP)

Answer: This change has been made

line 187: "was" not were fully enriched

Answer: This change has been made

line 305: remove "s" from differences

Answer: The “s” was removed

line 448: "in" not "into" glycosomes

Answer: “The” was removed

line 544: remove "the" before procyclic

Answer: “The” was removed
