# Supplementary material for: The glycosomal ATP-dependent phosphofructokinase of Trypanosoma brucei operates also in the gluconeogenic direction
Source: PLoS Biol. 2025 May 16;23(5):e3002938. doi: 10.1371/journal.pbio.3002938 (PMC12121924; doi:10.1371/journal.pbio.3002938)
Supplement: Attachment — Submitted filename: Response to Editor-v2.pdf [file pbio.3002938.s006.pdf]

a) We routinely suggest changes to titles to ensure maximum accessibility for a broad, non-specialist readership, and to ensure they reflect the contents of the paper. In this case, we would suggest a minor edit to the title, as follows. Please ensure you change both the manuscript file and the online submission system, as they need to match for final acceptance:

"The glycosomal ATP-dependent phosphofructokinase of Trypanosoma brucei operates in the gluconeogenic direction"

Answer: We consider that the proposed title is misleading, since it may suggest that PFK is only working in the gluconeogenic direction, which is not correct, since PFK works in the glycolytic direction in the presence of glucose. Consequently, we would prefer the following title:
"The glycosomal ATP-dependent phosphofructokinase of Trypanosoma brucei also operates in the gluconeogenic direction"

b) Please indicate what does the error bar indicate in Fig 8.

Answer: The error bar corresponds to the standard deviation value calculated from 3 independent biological replicates. We have included in the legend: "Each value corresponds to the mean of 3 independent biological replicates."

c) You may be aware of the PLOS Data Policy, which requires that all data be made available without restriction: http://journals.plos.org/plosbiology/s/data-availability. For more information, please also see this editorial: http://dx.doi.org/10.1371/journal.pbio.1001797
Please supply the numerical values either in a supplementary file or as a permanent DOI'd deposition for the following figures:
Figure 2, 3BCD, 4BC, 5DE, 7B, 8, 9CDIJ, S1
NOTE: the numerical data provided should include all replicates AND the way in which the plotted mean and errors were derived (it should not present only the mean/average values).

Answer: The file "Metadata-Plazolles et al 2025-v2" contains all the metadata.
Figure 2 (OK), 3BCD (OK), 4BC (OK), 5D (OK), 5E (OK), 7B (OK), 8 (OK), 9CDIJ (OK), S1 (OK)

d) Please cite the location of the data clearly in all relevant main and supplementary Figure legends, e.g. "The data underlying this Figure can be found in S1 Data" or "The data underlying this Figure can be found in https://doi.org/10.5281/zenodo.XXXXX"

Answer: The excel file containing all the numerical data, as well as the original, uncropped and minimally adjusted images (Metadata-Plazolles et al 2025-v2) is located at https//doi.org/10.5281/zenodo.15148560.

e) Please ensure that you are using best practice for statistical reporting and data presentation. These are our guidelines https://journals.plos.org/plosbiology/s/best-practices-in-research-reporting#loc-statistical-reporting and a useful resource on data presentation
https://journals.plos.org/plosbiology/article?id=10.1371/journal.pbio.1002128

- If you are reporting experiments where n ≤ 5, please plot each individual data point.

Answer: We have shown each individual data point in figures were 3C, 7B, 8, 9I-J the reporting experiments where n<5. However, this has not been applied to Figures 2, 3D and 4C, as the graphical representation of each individual data point would make these figures unreadable.

f) We require the original, uncropped and minimally adjusted images supporting all blot and gel results reported in the Figures 1B, 2, 3A, 4A, 5BC, 6BC, 7A, 9H, S2A
We will require these files before a manuscript can be accepted so please prepare and upload them now. Please carefully read our guidelines for how to prepare and upload this data:
https://journals.plos.org/plosbiology/s/figures#loc-blot-and-gel-reporting-requirements

Answer: All the uncropped and minimally adjusted images are in the excel file located at https//doi.org/10.5281/zenodo.15148560 (Metadata-Plazolles et al 2025-v2).

g) Please ensure that your Data Statement in the submission system accurately describes where your data can be found and is in final format, as it will be published as written there.

Answer: We made sure the location of the metadata is described in the Data Statement.

h) Per journal policy, if you have generated any custom code during the course of this investigation, please make it available without restrictions upon publication. Please ensure that the code is sufficiently well documented and reusable, and that your Data Statement in the Editorial Manager submission system accurately describes where your code can be found.
Please note that we cannot accept sole deposition of code in GitHub, as this could be changed after publication. However, you can archive this version of your publicly available GitHub code to Zenodo. Once you do this, it will generate a DOI number, which you will need to provide in the Data Accessibility Statement (you are welcome to also provide the GitHub access information). See the process for doing this here: https://docs.github.com/en/repositories/archiving-a-github-repository/referencing-and-citing-content

Answer: We have not generated ant custom code

1) Please send a completed 'Competing Interests' statement, including any COIs declared by your co-authors. If you have no competing interests to declare, please state "The authors have declared that no competing interests exist". Otherwise please declare all competing interests beginning with the statement "I have read the journal's policy and the authors of this manuscript have the following competing interests:"

Answer: We have included the sentence "The authors have declared that no competing interests exist" in the 'Competing Interests' statement.

2) Your current Financial Disclosure states, " FB was supported by the Centre National de la Recherche Scientifique (CNRS, https://www.cnrs.fr/), the Université de Bordeaux (https://www.u-bordeaux.fr/), the Agence National de Recherche (ANR, https://anr.fr/) ADIPOTRYP (grant number ANR-19-CE15-0004-01) and TRYPADIFF (grant number ANR-23-CE15-0040-01), the Laboratoire d'Excellence (https://www.enseignementsup-recherche.gouv.fr/cid51355/laboratoires-d-excellence.html) through the LabEx ParaFrap (grant number ANR-11-LABX-0024), and the "Fondation pour le Recherche Médicale" (FRM) ("Equipe FRM", grant n̊ EQU201903007845). The funders had no role in study design, data collection and analysis, decision to publish, or preparation of the manuscript.".
However, your funding information on the submission form indicates that you did not receive funding. Please indicate by return email the full and correct funding information for your study and confirm the order in which funding contributions should appear. Please be sure to indicate whether the funders played any role in the study design, data collection and analysis, decision to publish, or preparation of the manuscript.

Answer: A message was sent on the 31 mars 2025 to PLoS Biol editorial manager to state that we indeed received funding and the above statement is correct.

Funder	Grant number	Recipient
Centre National de la Recherche Scientifique		FB
the Université de Bordeaux		FB
the Agence National de Recherche		FB
ADIPOTRYP	ANR-19-CE15-0004-01	FB
TRYPADIFF	ANR-23-CE15-0040-01	FB
the Laboratoire d'Excellence	ANR-11-LABX-0024	FB
"Fondation pour le Recherche Médicale"	?EQU201903007845	FB

3) We do not publish any copyright or trademark symbols that usually accompany proprietary names, eg (R), (C), or TM (e.g. next to drug or reagent names). Please remove all instances of trademark/copyright symbols throughout the text, including XcellTM on line no 812, FACSAriaTM on line no 955

Answer: “TM” were removed

4) We notice that your supplementary figures are uploaded with the file type 'Figure'. Please amend the file type to 'Supporting Information'. Please ensure that each Supporting Information file has a legend listed in the manuscript after the references list.

Answer: Same than 5)

5) We notice that your supplementary figures are included in the manuscript file. Please remove them and upload them with the file type 'Supporting Information'. Please ensure that each Supporting Information file has a legend listed in the manuscript after the references list.

Answer: Legends included after the reference list

Please refer to the following article for PLOS Biology's best-practice recommendations for data presentation: https://journals.plos.org/plosbiology/article?id=10.1371/journal.pbio.1002128.
Guidelines for statistical reporting, if relevant, may be found here:
https://journals.plos.org/plosbiology/s/best-practices-in-research-reporting#loc-statistical-reporting.
